# Why Can't We Accurately Predict Others' Decisions? Prediction Discrepancy in Risky Decision-Making

**DOI:** 10.3389/fpsyg.2018.02190

**Published:** 2018-11-13

**Authors:** Qingzhou Sun, Huanren Zhang, Jing Zhang, Xiaoning Zhang

**Affiliations:** ^1^College of Economics and Management, Zhejiang University of Technology, Hangzhou, China; ^2^Department of Business and Economics, University of Southern Denmark, Odense, Denmark

**Keywords:** predictor, actor, prediction discrepancy, risk preferences, anticipated emotion

## Abstract

Individuals often fail to accurately predict others' decisions in a risky environment. In this paper, we investigate the characteristics and causes of this prediction discrepancy. Participants completed a risky decision-making task mixed with different domains (gain vs. loss) and probabilities (small vs. large), with some participants making decisions for themselves (the actor) and the others predicting the actors' decisions (the predictor). The results demonstrated a prediction discrepancy: predictions were more risk-averse than the actual decisions over small-probability gains and more risk-seeking over large-probability gains, while these patterns were reversed in the loss domain. Reported and predicted levels of emotional stimulation revealed a pattern that is consistent with the notion of risk-as-feelings and empathy gaps. Mediation analysis provided strong evidence that such prediction discrepancy is driven mainly by the predictor's underestimate of the intensity (not the impact) of the actor's emotional state.

## Introduction

*If you know yourself and know the enemy, you are bound to win in all battles*.

*- Sun Tzu, The Art of War*

Because virtually all real-world decisions involve risk, it is important to predict the risk preferences of others in order to make optimal decisions in social and economic interactions (Hsee and Weber, 1997; Faro and Rottenstreich, 2006; Kurt and Inman, 2012). For example, the disputing parties in litigation need to appreciate each other's risk preferences to increase their chance of winning a lawsuit or reaching a settlement; a firm needs to accurately evaluate the risk preference of its competitor's CEO in order to decide whether it is optimal to make a risky investment in developing a new product; insurance companies need to assess customers' risk preferences in order to design optimal insurance packages and increase their sales; employers should recognize their employees' risk preferences in order to implement a payment scheme that induces the best performance; and legislators need to appraise people's risk preferences in order to design laws that effectively deter misfeasance and crime. Accurate prediction of risk preferences is also important when one needs to make decisions for others: a broker, for example, needs to understand the customer's risk preferences to come up with the best investment portfolio, while a physician needs to comprehend the patient's risk preferences in order to implement a treatment that is based tailored to that patient's needs.

Unfortunately, individuals often fail to accurately predict others' preferences and decisions, giving rise to a prediction discrepancy (Hsee and Weber, 1997; Van Boven and Loewenstein, 2003). It is vital to understand the factors that cause and influence this prediction discrepancy in order to help people make better decisions. Although a small body of literature has studied prediction discrepancy, the findings have been rather inconsistent. Some studies have observed that the predictions are more risk-seeking than the actual decisions (e.g., Hsee and Weber, 1997; Krishnamurthy and Kumar, 2002; Eckel and Grossman, 2008, Study 1; Sun et al., 2017b), whereas others reported the opposite findings (e.g., Krishnamurthy and Kumar, 2002; Fernandez-Duque and Wifall, 2007, Study 2a; Levinger and Schneider, 1969). Notably, these studies involve different conditions: some involve decision-making over gains (Hsee and Weber, 1997; Krishnamurthy and Kumar, 2002, Study 1), while others involve decision-making in the loss domain (Krishnamurthy and Kumar, 2002; Fernandez-Duque and Wifall, 2007, Study 2a); some involve small-probability events (Fernandez-Duque and Wifall, 2007), whereas others involve large-probability events (Hsee and Weber, 1997; Eckel and Grossman, 2008; Sun et al., 2017a). This suggests that such prediction discrepancy might be modulated by the domain (gain vs. loss) and the probability (small vs. large).

In this paper, we use an experiment to investigate the characteristics and the causes of this prediction discrepancy in risky decision-making. We aim to reconcile the conflicting findings listed above by providing a systematic study that involves different domains and probabilities.

### Pattern of risk preferences

Empirical studies have demonstrated a robust four-fold pattern of risk preferences when people make decisions under different domains and probabilities: (1) risk-seeking over small-probability gains; (2) risk-averse over large-probability gains; (3) risk-averse over small-probability losses; and (4) risk-seeking over large-probability losses (Tversky and Kahneman, 1992; Fox and Poldrack, 2009). For example, when facing a choice between “a 1% chance of winning $200” and “a 100% chance of winning $2,” people tend to choose the former, riskier, option; between “a 99% chance of winning $200” and “a 100% chance of winning $198,” people tend to choose the latter, risk-free, option. Conversely, when asked to choose between “a 1% chance of losing $200” and “a 100% chance of losing $2,” people tend to choose the latter, risk-free, option; between “a 99% chance of losing $200” and “a 100% chance to lose $198,” people tend to choose the former, riskier, option.

The notion of risk-as-feelings (Loewenstein et al., 2001) provides an explanation for this four-fold pattern: people often experience emotional reactions to risk and make choices that are driven partially by *anticipated emotions* (Rottenstreich and Hsee, 2001; Brandstätter et al., 2002; Kliger and Levy, 2008; Tyszka and Sawicki, 2011; Suter et al., 2015). With a small probability of potential gains/losses, the status quo may serve as a salient reference point; compared to this status quo, the possibility of a gain/loss stimulates elation/disappointment. In contrast, with a large probability, the salient reference point may be the potential gain/loss, and the possibility of an unrealized gain/loss can result in disappointment/elation. In other words, with small-probability gains or large-probability losses, the *anticipated elation* after having won a very unlikely prize or having avoided a very likely loss makes people risk-seeking; with large-probability gains or small-probability losses, the *anticipated disappointment* after having failed to win a very likely prize or having suffered from a very unlikely loss makes people risk-averse (Brandstätter et al., 2002). Consistent with the notion of risk-as-feelings, this four-fold pattern of risk preferences has been shown to be more pronounced when the anticipated emotions become more intense (Rottenstreich and Hsee, 2001; Kliger and Levy, 2008; Suter et al., 2015).

### Predicting the decisions of others

People often fail to accurately predict others' preferences and decisions in many situations, such as product valuations (Kurt and Inman, 2012), driving behaviors (Svenson, 1981), and medical decision-making (Garcia-Retamero and Galesic, 2012). Studies have suggested that the empathy gap between the predictor and the actor plays an important role in generating this prediction discrepancy—that is, a predictor may underestimate the intensity and impact of the actor's emotional state (Loewenstein, 1996; Van Boven and Loewenstein, 2003; Faro and Rottenstreich, 2006; Williams et al., 2014). For instance, Williams et al. (2014) report that people predicting the decisions of others experienced less anticipated elation in response to positive events and less anticipated disappointment in response to negative events than those who made decisions for themselves. Likewise, recent neuroimaging studies observed that the amygdala, a key neural structure related to emotional activity (Morrison and Salzman, 2010), was less active when people tried to predict others' decisions than when they made decisions for themselves (Albrecht et al., 2010; Jung et al., 2013).

These studies suggest that anticipated emotions are less intense for the predictor than they are for the actor. Therefore, we hypothesize that, due to the less intense anticipated emotion experienced by the predictor, the prediction would exhibit an attenuated four-fold pattern. That is:

***Hypothesis 1*** Individuals predict others' decisions to be more risk-neutral than they actually are.

Hypothesis 1 can be tested in four different situations: (1) In small-probability gains, an individual would predict others to be less risk-seeking; (2) in small-probability losses, an individual would predict others to be less risk-averse; (3) in large-probability gains, an individual would predict others to be less risk-averse; and (4) in large-probability losses, an individual would predict others to be less risk-seeking.

The empathy gap can influence the prediction discrepancy through two possible channels: (a) underestimation of the *intensity* of the emotional state; and (b) underestimation of the *impact* of the emotional state (Loewenstein, 1996; Van Boven et al., 2000). The second channel can influence prediction discrepancy because, even if predictors correctly predict the intensity of the actors' emotional state, they may underestimate how much that emotional state would impact the actors' decisions. While previous studies have demonstrated the effect of the empathy gap, they do not examine the relative importance of these two channels. We propose the following two hypotheses related to the effect of the empathy gap:

***Hypothesis 2*** The prediction discrepancy is caused by the predictors' underestimate of the *intensity* of the actors' emotional state.

***Hypothesis 3*** The prediction discrepancy is caused by the predictors' underestimate of the *impac*t of the actors' emotional state.

In a related study, Faro and Rottenstreich (2006) provide some initial evidence for patterns of the prediction discrepancy that are consistent with our Hypothesis 1. Using a pricing task, participants were asked to indicate the amount of cash that made them indifferent between receiving the cash and playing a lottery with a certain probability (0.001 or 0.99) of winning $4000. The results showed that the predictions were more risk-neutral than the choices. Further, they found that participants with high levels of self-reporting empathy made more-accurate predictions.

There are some important questions that Faro and Rottenstreich (2006) did not explore. First, they investigated only the effect of empathy gaps in the gain domain and did not distinguish the effects of different types of emotions on the prediction discrepancy. Second, without a direct measure of emotions, they could not compare the relative importance of the two channels (intensity underestimation vs. impact underestimation) through which the empathy gap can work. Third, they used only lotteries with extreme probabilities (0.001 and 0.99). As studies have suggested that extreme probabilities are not well-behaved in the probability weighting function (Kahneman and Tversky, 1979), it is important to investigate whether Faro and Rottenstreich's conclusions can be extended to less extreme probabilities. Finally, Faro and Rottenstreich (2006) used pricing tasks to measure risk preferences—that is, participants were asked to indicate their certainty equivalents for lotteries. Many studies have shown that pricing tasks and choice tasks (choosing between a lottery and a certain amount of cash) could lead to drastically different measures of risk preferences (Harbaugh et al., 2010). Therefore, it is unclear whether we would observe the same pattern of prediction discrepancies in choice tasks.

By answering the aforementioned questions, our study greatly complements, and extends Faro and Rottenstreich's 2006 study. In our experiment, participants were asked to complete a series of choice tasks that involve a wide range of probabilities in both the loss and gain domains. By directly eliciting emotional intensities, we investigated the correlation between emotional states and prediction discrepancies, which can shed light on the relative importance of the two channels.

## Experiment design

One hundred and thirty-eight undergraduates (62 women; *M*_*age*_ = 22.12 years, *SD* = 1.89 years) participated in the experiment^1^. Participants were randomly assigned as either an actor who made decisions for him/herself (*N* = 69, 34 women; *M*_*age*_ = 22.52 years, *SD* = 1.67 years), or a predictor who predicted others' decision (*N* = 69, 33 women; *M*_*age*_ = 22.13 years, *SD* = 1.35 years). Regardless of their roles, participants completed a series of decision problems mixed with different domains (gain vs. loss) and probabilities (small vs. large). Therefore, we had the decision maker's role as a between-participant variable^2^ and the domain and the probability as within-participant variables. The dependent variable was risk preference. This study was carried out in accordance with the recommendations of Ethics Committee of East China Normal University with written informed consent from all subjects. All subjects gave written informed consent in accordance with the Declaration of Helsinki. The protocol was approved by the Ethics Committee of East China Normal University.

The experiment consisted of 7 experimental sessions, each session with around 20 participants. In each session, participants were randomly assigned to a computer upon arriving at the laboratory. Each session had an even number of participants, and half of them were randomly selected as the predictors, and the other half as the actors. Participants were told that they would see the same set of decision problems on the computer screen, one making decisions for him/herself and the other predicting these decisions. After they had completed the tasks, the predictors were randomly matched with the actors to determine the accuracy of their predictions. The participants were not allowed to communicate or interact with one another during the experiment.

**Figure 1:**
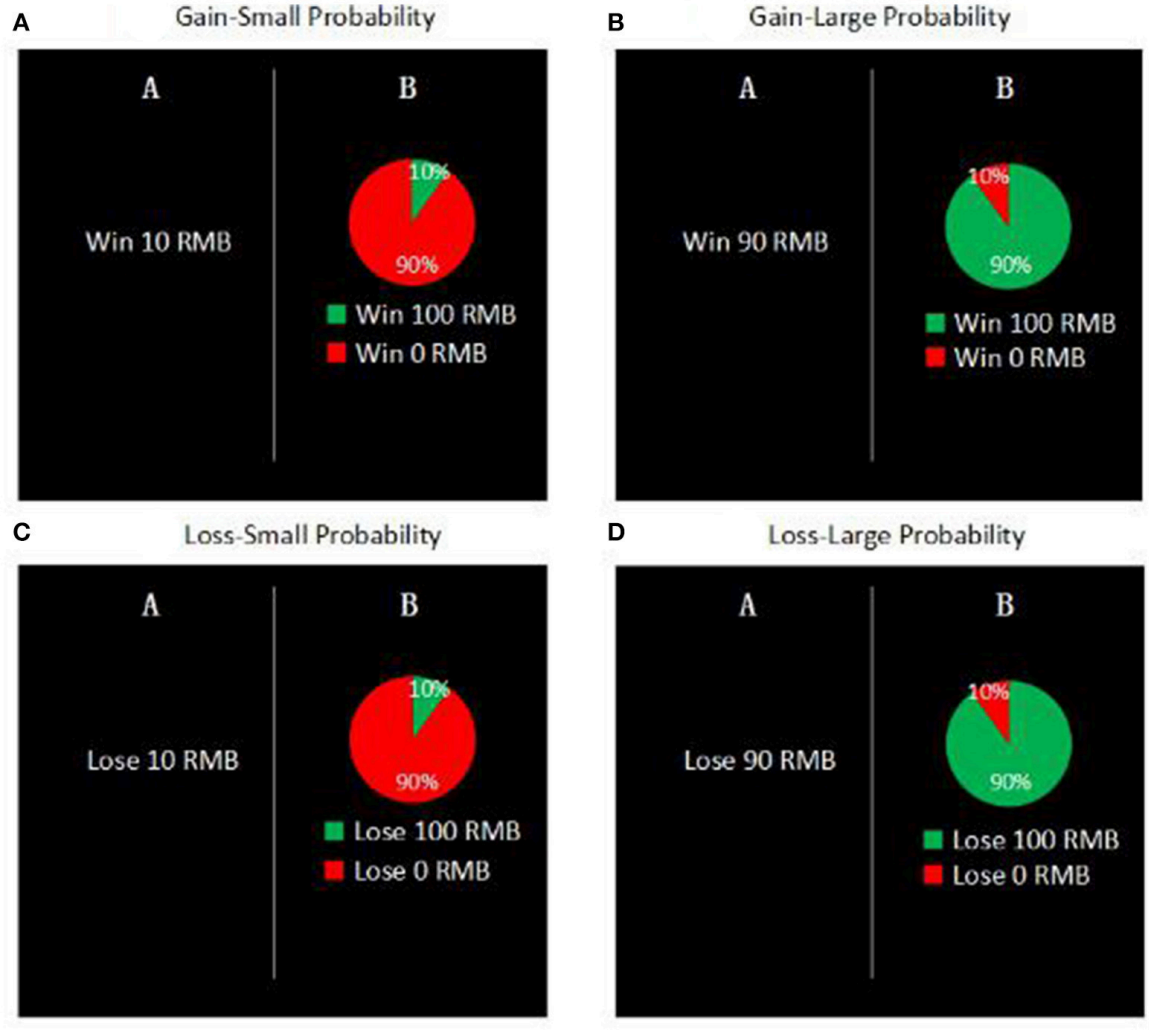
Examples of the decision problems.

Each participant completed a series of 90 decision problems on the computer for measuring their risk preferences (De Martino et al., 2006). Each decision problem included two options. One was a risky option (i.e., with a probability of *p*_*i*_ to obtain RMB *x*_i_ and a probability of (1–*p*_i_) to obtain nothing), whereas the other was a risk-free option (i.e., to obtain RMB *z*_*i*_ for sure)^3^. In different decision problems, *p*_*i*_ took the value of 1, 5, 10, 25, 50, 75, 90, 95, or 99%, while *x*_*i*_ took the value of ±50, ±100, ±200, ±400, or ±800. The two options had the identical expected value (i.e., *p*_*i*_ × *x*_i_ + (1–*p*_i_) × 0 = _*i*_). As shown in Figure 1, a pie chart was used to depict the probability of winning or losing along with the numerical values (De Martino et al., 2006). After seeing the chart, participants were first asked to indicate their or their partner's anticipated emotion after each choice: how elated they (or their partner) would feel if choosing the risky option and reaching the desired outcome (1 = *not at all*; 9 = *extremely*); how disappointed they (or their partner) would feel if choosing the risky option and reaching the undesired outcome (1 = *not at all*; 9 = *extremely*) (see Brandstätter et al., 2002). After that, participants made a choice between the two options.

The order of the decision problems was counterbalanced across the pairs. After the decision task, the participants were asked to identify their roles in the decision-making as a manipulation check (0 = *predicting the decisions of others*; 1 = *making decisions for the self*) and to complete control variable measures, including their perceived difficulties of the tasks; efforts exerted in performing the tasks; confidence in their decisions, made on 9-point scales (1 = *not at all*, 9 = *very much*); and demographic information (i.e., gender and age). Finally, the participants were thanked, debriefed, and paid RMB25 as the participation fee.

## Results

### Manipulation checks

Five participants, all of whom were predictors, failed the manipulation check: they reported that they had been making decisions for themselves instead of predicting the decisions of others. Thus, we excluded them from the analysis. The actors and predictors did not differ from one another in terms of gender, χ^2^(1, *N* = 133) = 0.41, *p* = 0.523, and age *t*_(131)_ = 1.50, *p* = 0.137, Cohen's *d* = 0.23, respectively.

### Calculation of the risk-preference index

Following previous studies (Hsee and Weber, 1997; Krishnamurthy and Kumar, 2002), we calculated the proportion of risky options among each participant's choices to serve as the risk preference (*RP*) index^4^. The *RP* index had a range between 0 and 1. Risk-neutral individuals should have an RP index close to 0.5, while larger values of RP index indicate stronger risk-seekingness. To simplify the analysis and to avoid the complication presented by the correlation between decisions by the same participant, we divided the decisions into four conditions (small probability gain, small probability loss, large probability gain, and large probability loss). Following Tversky and Kahneman (1992), we defined *p* < 0.5 as small probability and *p* ≥ 0.5 as large probability^5^. For each condition, we calculated the proportion of risky choices for each participant as the individual RP index under that condition.

### Risk preferences and anticipated emotions

Table 1 provides the statistics of the risk preferences and anticipated emotions for both the actors and the predictors, separately for the four different conditions. The level of anticipated emotion is normalized so that its value is between 0 and 1. To assess whether there were prediction discrepancies in risk preference, we performed a 2(decision maker's role) × 2(domain) × 2 (probability) analysis of variance (ANOVA) on the *RP* index and found a main effect for probability, *F*_(1, 131)_ = 92.88, *p* < 0.001, η_*p*_^2^ = 0.42, such that the participants were more risk-seeking in large-probability condition (*M* = 0.49, *SD* = 0.14) than in small-probability condition (*M* = 0.40, *SD* = 0.13). Consistent with cumulative prospect theory (Tversky and Kahneman, 1992), we also obtained an interaction between domain and probability, *F*_(1, 131)_ = 633.09, *p* < 0.001, η_*p*_^2^ = 0.83. In small-probability condition, the participants were more risk-seeking in the gain domain (*M* = 0.53, *SD* = 0.18) than in the loss domain (*M* = 0.27, *SD* = 0.15), *F*_(1, 131)_ = 247.80, *p* < 0.001, η_*p*_^2^ = 0.65, whereas in large-probability condition, the participants were more risk-seeking in the loss domain (*M* = 0.62, *SD* = 0.16) than in the gain domain (*M* = 0.35, *SD* = 0.16), *F*_(1, 131)_ = 371.30, *p* < 0.001, η_*p*_^2^ = 0.92, which suggests that both choices and predictions exhibit the four-pattern risk preferences^6^.

**Table 1:** Mean (± *SD*) RP index and anticipated e motions.

		**RP index**			**Elation**			**Disappointment**		
		**Actor**		**Predictor**	**Actor**		**Predictor**	**Actor**		**Predictor**
Small probability	Gain	0.58 (0.18)	>^***^	0.47 (0.16)	0.58 (0.27)	>^**^	0.45 (0.23)	0.19 (0.20)	>	0.15 (0.11)
	Loss	0.23 (0.13)	<^***^	0.32 (0.15)	0.20 (0.16)	<	0.22 (0.16)	0.54 (0.25)	>^*^	0.44 (0.24)
Large probability	Gain	0.31 (0.15)	<^**^	0.39 (0.17)	0.25 (0.18)	<	0.23 (0.18)	0.60 (0.24)	>^**^	0.46 (0.21)
	Loss	0.66 (0.15)	>^**^	0.58 (0.15)	0.50 (0.25)	>^***^	0.30 (0.22)	0.24 (0.19)	<	0.25 (0.15)

Statistical significance is based on analysis of variance.

*** p < 0.001,

** p < 0.01,

* *p < 0.05*.

Crucially, we observed an interaction among decision maker's role, domain and probability (Table 1), *F*_(1, 131)_ = 77.98, *p* < 0.001, η_*p*_^2^ = 0.37. In small-probability gains, the participants who predicted decisions of others (*M* = 0.47, *SD* = 0.16) were less risk-seeking than those who made decisions for themselves (*M* = 0.58, *SD* = 0.18), *F*_(1, 131)_ = 14.21, *p* < 0.001, η_*p*_^2^ = 0.10, whereas in large-probability gains, the participants who predicted decisions of others (*M* = 0.39, *SD* = 0.17) were less risk-averse than those who made decisions for themselves (*M* = 0.31, *SD* = 0.15), *F*_(1, 131)_ = 7.80, *p* = 0.006, η_*p*_^2^ = 0.06. Conversely, in small-probability losses, the participants who predicted decisions of others (*M* = 0.32, *SD* = 0.15) were less risk-averse than those who made decisions for themselves (*M* = 0.23, *SD* = 0.13), *F*_(1, 131)_ = 14.71, *p* < 0.001, η_*p*_^2^ = 0.10, whereas in large-probability losses, the participants who predicted decisions of others (*M* = 0.58, *SD* = 0.15) were less risk-seeking than those who made decisions for themselves (*M* = 0.66, *SD* = 0.15), *F*_(1, 131)_ = 9.21, *p* = 0.003, η_*p*_^2^ = 0.07. The prediction discrepancies are consistent with our hypotheses: predictors are significantly more risk-neutral (*RP* index closer to 0.5) under all four conditions. No other main effects and interactions were significant, *ps* > 0.16.

Looking at Table 1 for the four different conditions, we can notice that the prediction discrepancy is highly correlated with the difference in anticipated emotion. Note that the actual and predicted levels of elation from reaching the desired outcome under small probability loss and large probability gain, as well as the actual and predicted levels of disappointment from reaching the undesired outcome, are not statistically significant–these levels of anticipated emotion can be considered as the baseline level. In contrast, the anticipated emotion in all other situations demonstrates a significant difference: predictors do not fully appreciate the anticipated elation experienced by the actors for small probability gains [*F*_(1, 131)_ = 8.46, *p* = 0.004, η_*p*_^2^ = 0.06] and large probability losses [*F*_(1, 131)_ = 23.04, *p* < 0.001, η_*p*_^2^ = 0.15]; on the other hand, predictors fail to fully appreciate the anticipated disappointment experienced by the actors for small probability losses [*F*_(1, 131)_ = 5.96, *p* = 0.016, η_*p*_^2^ = 0.04] and large probability gains [*F*_(1, 131)_ = 12.44, *p* = 0.001, η_*p*_^2^ = 0.09].

The difference between actual and predicted emotion intensity demonstrates an empathy gap between the actor and the predictor (Loewenstein, 1996). While predictors correctly predict the type of emotional state the actors would experience, they underestimate its *intensity*. The empathy gap can aggravate the prediction discrepancy if predictors underestimate the *impact* of the emotional state—given an equally intense emotional state, the predictors underestimate the actors' reaction to it.

### Two channels of empathy gaps

We can use the following regressions to understand the connection between the anticipated emotion and the prediction discrepancy and to disentangle the two channels through which the empathy gap can work.

(1) $$\begin{array}{l} RP\;index=\beta_{0}^{1}+\beta_{1}^{1}Predictor. \end{array}$$

(2) $$\begin{array}{l} RP\;index=\beta_{0}^{2}+\beta_{1}^{2}Predictor+\beta_{2}^{2}Elation\\ \;+\beta_{3}^{2}Disappointment \end{array}$$

(3) $$\begin{array}{l} RP\;index=\beta_{0}^{3}+\beta_{1}^{3}Predictor+\beta_{2}^{3}Elation\\ \;+\beta_{3}^{3}Disappointment+\beta_{4}^{3}Predictor\;\times Elation\\ \;+\beta_{5}^{3}Predictor\times Disappointment \end{array}$$

Equation (1) is a simple regression of the *RP index* on the dummy variable *Predictor*, which takes 1 for the predicted values and 0 otherwise. If a prediction discrepancy exists, we would expect $\beta_{1}^{1}$ to be statistically significant. Equation (2) includes anticipated emotions as explanatory variables. According to the notion of risk-as-feelings, we would expect $\beta_{2}^{2}$ to be significantly positive and $\beta_{2}^{2}$ to be significantly negative. If the prediction discrepancy is mainly caused by predictors underestimating the intensity, we would have an insignificant $\beta_{1}^{2}$. Equation (2) investigates how the underestimate of emotional intensity influences prediction discrepancy, however, it does not take into consideration the possibility that predictors can underestimate the impact of the emotions, even if they accurately estimate the actors' emotional intensity. The two interactions terms (with coefficients $\beta_{4}^{3}\;and\;\beta_{5}^{3}$) in Equation (3) allow us to investigate whether being a predictor moderates the effect of emotional states—that is, whether the predictors underestimate the *impact* of emotional states. If the prediction discrepancy can be attributed, in part, to the underestimated impact of emotional states, $\beta_{4}^{3}\;$would be statistically negative and $\beta_{5}^{3}$ statistically positive.

Table 2 shows the regression results separately for each of the four conditions, controlling for the effect of gender and age. Models (1), (3), (5), and (7) provide results that are consistent with the statistic tests shown in Table 1. Models (2), (5), (8), and (11) investigate the effect of predictors underestimating emotional intensity on the prediction discrepancy. Note that the coefficient of “predictor” decreases significantly after including emotional intensity as explanatory variables, demonstrating that the underestimate of emotional intensity by the predictors is an important factor causing the prediction discrepancy.

**Table 2:** Regression analysis on RP index under four different conditions.

	**Small-probability gain**			**Small-probability loss**			**Large-probability gain**			**Large-probability loss**		
	**(1)**	**(2)**	**(3)**	**(4)**	**(5)**	**(6)**	**(7)**	**(8)**	**(9)**	**(10)**	**(11)**	**(12)**
Predictor	−0.12^***^	−0.08^***^	−0.05	0.09^***^	0.06^***^	0.02	0.07^**^	0.04	0.10	−0.09^***^	−0.02	−0.12
	(0.03)	(0.02)	(0.07)	(0.03)	(0.02)	(0.05)	(0.03)	(0.03)	(0.07)	(0.03)	(0.03)	(0.08)
Elation		0.35^***^	0.36^***^		0.15^*^	0.11		0.22^***^	0.18^*^		0.30^***^	0.22^***^
		(0.05)	(0.07)		(0.09)	(0.10)		(0.07)	(0.10)		(0.05)	(0.08)
Disappointment		−0.13	−0.12		−0.18^***^	−0.20^***^		−0.22^***^	−0.16^**^		−0.11	−0.17
		(0.09)	(0.10)		(0.05)	(0.06)		(0.05)	(0.06)		(0.08)	(0.12)
Predictor × Elation			−0.03			0.08			0.07			0.16
			(0.11)			(0.18)			(0.14)			(0.11)
Predictor × Disappointment			−0.03			0.05			−0.14			0.13
			(0.19)			(0.09)			(0.11)			(0.16)
Male	0.06^*^	0.05^*^	0.04^*^	0.04	0.03	0.03	0.05^*^	0.05^**^	0.05^**^	0.01	0.01	0.01
	(0.03)	(0.02)	(0.02)	(0.02)	(0.02)	(0.02)	(0.03)	(0.03)	(0.03)	(0.03)	(0.02)	(0.02)
Age	−0.01	−0.01	−0.01	−0.01^**^	−0.01^**^	−0.01^**^	−0.01	−0.01	−0.01	−0.01	−0.01	−0.01
	(0.01)	(0.01)	(0.01)	(0.01)	(0.01)	(0.01)	(0.01)	(0.01)	(0.01)	(0.01)	(0.01)	(0.01)
Constant	0.80^***^	0.56^***^	0.54^***^	0.54^***^	0.61^***^	0.64^***^	0.42^**^	0.56^***^	0.51^***^	0.83^***^	0.66^***^	0.76^***^
	(0.20)	(0.19)	(0.19)	(0.15)	(0.15)	(0.15)	(0.18)	(0.18)	(0.18)	(0.19)	(0.19)	(0.21)
Observations	133	133	133	133	133	133	133	133	133	133	133	133
R-squared	0.13	0.39	0.39	0.14	0.24	0.25	0.08	0.23	0.24	0.08	0.30	0.32

Robust standard errors in parentheses.

*** p < 0.001,

** p < 0.01,

* *p < 0.05*.

Models (3), (6), (9), and (12) further include the interaction terms between the decision maker's role and the intensity of the anticipated emotions, capturing the effect of predictors' underestimating the emotional impact. The significance of the emotional intensity (coefficients of the variable “elation” and “disappointment”) is not influenced by the including these interaction terms. After controlling for the effect of the emotional state (both the intensity and the impact), action, and prediction do not show a significant difference, implying that the prediction discrepancy can be explained by the empathy gap. In all four models, the anticipated elation of reaching the desired outcome of the risky option makes people significantly more risk-seeking, while the anticipated disappointment of reaching the undesired outcome makes people significantly more risk-averse (*p* < 0.01). However, the effect of the anticipated disappointment is significant only for small-probability loss and large-probability gain, and the effect of the anticipated elation is significant only for small-probability gain and large-probability loss (marginally significant for large-probability gain).

These models also allow us to disentangle the two channels through which the empathy gap can influence prediction discrepancy. If the predictor underestimates the impact of the emotional state on the actor's choice, we would expect a moderating effect of the participant's role on the emotional state. However, the interactions between anticipated emotion and the participant's role are not significant, and a joint *F*-test of these two interaction terms is also insignificant, indicating that the effect of emotional intensity is not significantly moderated by participant's role. This provides strong evidence that the prediction discrepancy is caused primarily by the underestimation of the *intensity* (not the *impact*) of the emotional state^7^.

Table 2 also shows that male participants are slightly more risk-seeking, while older participants tend to be more risk-averse. However, these effects are small in size and not always statistically significant across different conditions. The regression results are almost identical if these two variables are excluded.

**Figure 2:**
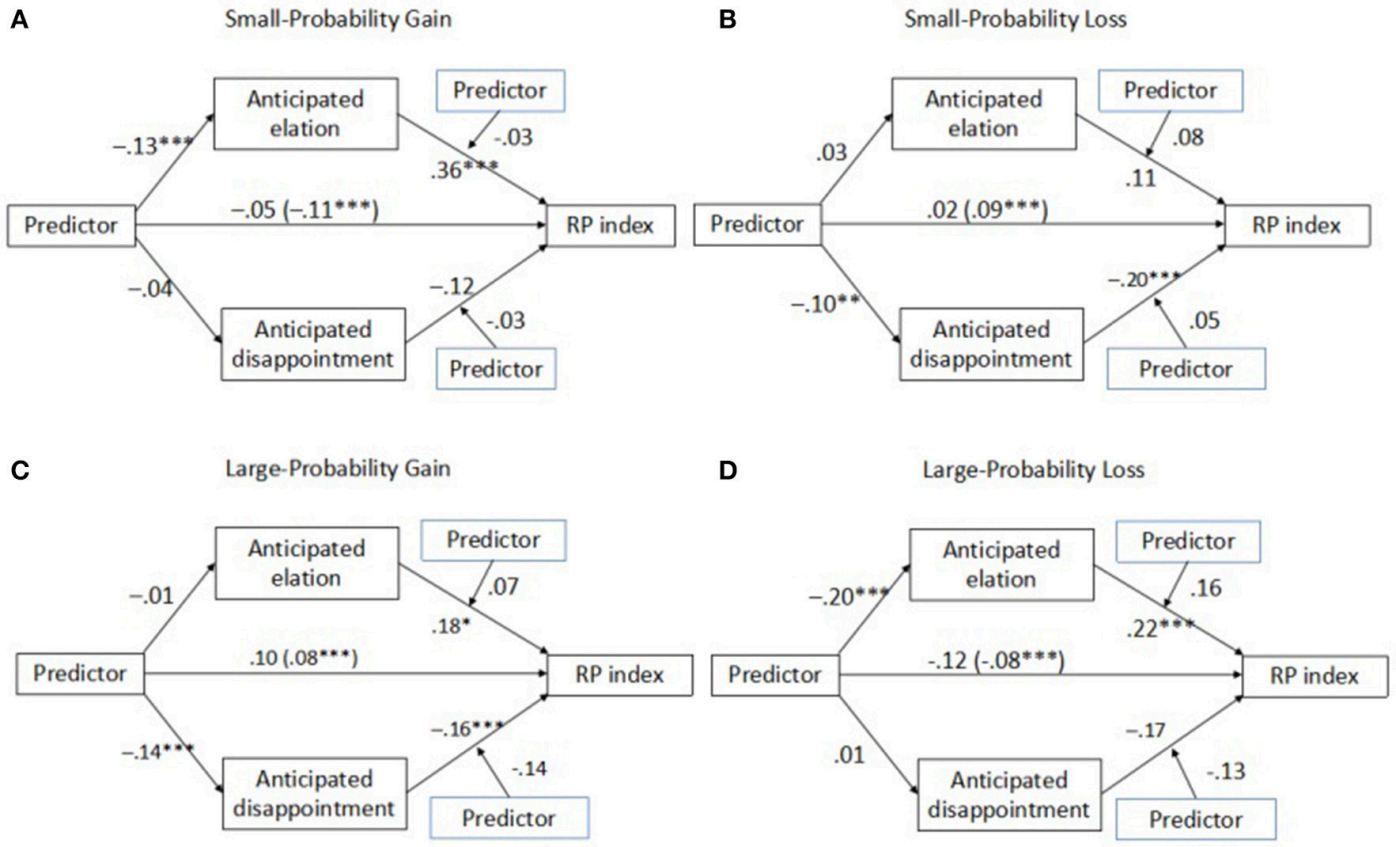
Path analysis demonstrates that the prediction discrepancy is driven mainly by the underestimate of the intensity, not the impact, of the emotional state by the predictor. For the path from *Predictor* to *RP index*, the coefficient in the parentheses represents the regression coefficient without controlling for the effects of anticipated emotion. All path coefficients represent unstandardized regression weights after controlling the effects of age and gender. ^***^p < 0.001, ^**^p < 0.01, *p < 0.05.

Figure 2 and Table 3 provide the path analysis that visualizes the mediating effects of the anticipated emotions on the prediction discrepancy and the relative importance of the two channels of empathy gaps. In each condition, the participant's role (actor = 0, predictor = 1) was treated as the independent variable and a potential moderator for the anticipated emotions. Anticipated elation and disappointment were treated as potential mediators. We used the bootstrapping procedure from Hayes and Preacher (2014) and the corresponding SPSS PROCESS macro to test for mediated moderation. The analysis shows that the prediction discrepancies were mediated mainly by the anticipated elation for small-probability gains and large- probability losses, as well as by the anticipated disappointment for small-probability losses and large-probability gains (Table 3). In the four models, the results showed no moderating effect of the decision-maker's role in the relationship between anticipated emotions and risk preferences, further suggesting that the primary cause of the prediction discrepancy is the underestimate of the *intensity* (not the *impact*) of the emotional state.

**Table 3:** Direct and indirect effects in the path analysis.

		**Direct effect**	**Mediating effects**		**Moderating effects**	
		**Predictor**	**Anticipated elation**	**Anticipated disappointment**	**Anticipated elation**	**Anticipated disappointment**
Small-probability gain	Bootstrap estimate(*±SE*)	−0.08(0.03)	−0.05(0.02)	0.01(0.01)	−0.04(0.10)	−0.03(0.18)
	95% CI	[−0.13, −0.03]	[−0.08, −0.02]	[−0.01, 0.02]	[−0.23, 0.16]	[−0.39, 0.32]
	Ratio in total effect	63%	42%	−5%	–	–
Small-probability Loss	Bootstrap estimate(*±SE*)	0.06(0.2)	0.01(0.01)	0.02(0.01)	0.08(0.14)	0.06(0.10)
	95% CI	[0.02, 0.11]	[−0.01, 0.02]	[0.01, 0.04]	[−0.20, 0.37]	[−0.13, 0.24]
	Ratio in total effect	76%	4%	20%	–	–
Large-probability gain	Bootstrap estimate(*±SE*)	0.04(0.03)	−0.01(0.01)	0.03(0.01)	0.07(0.14)	−0.14(0.11)
	95% CI	[0.01, 0.10]	[−0.02, 0.01]	[0.01, 0.05]	[−0.21, 0.36]	[−0.37, 0.08]
	Ratio in total effect	65%	−5%	40%	–	–
Large-probability loss	Bootstrap estimate(*±SE*)	−0.02(0.03)	−0.06(0.01)	−0.01(<0.01)	0.16(0.10)	0.13(0.15)
	95% CI	[−0.07, −0.01]	[−0.10, −0.03]	[−0.01, 0.01]	[−0.05, 0.36]	[−0.16, 0.42]
	Ratio in total effect	25%	73%	2%	–	–

*Parallel multiple mediation analyses were conducted using the PROCESS macro (bootstrapping, 5,000 samples) for SPSS 18.0. CI, confidence interval. The numbers represent unstandardized bootstrap estimate*.

## Discussion

This study examined the discrepancy between the predictions about others' decisions and their actual decisions—a discrepancy commonly observed in daily life. Our results showed that predictions are more risk-neutral than the actual choices under all four conditions (small-probability gains, large-probability gains, small-probability losses, and large-probability losses). We find evidence that this discrepancy is driven mainly by the predictor's underestimate of the intensity of the actor's emotional state (i.e., anticipated elation and disappointment).

Prior studies have reached conflicting conclusions about prediction discrepancy in risky decision-making. For instance, Krishnamurthy and Kumar (2002) found that, in a task involving waiting time decisions in the gain domain, people who predicted others' decisions were more risk-seeking than those who actually made the decisions. However, Zhang et al. (2017) observed the opposite in a task involving loss situations. Hsee and Weber (1997) found that people made riskier choices in predicting others' decisions than in deciding for themselves when facing a decision between a 50% chance to win big cash and a 100% chance to win small cash, whereas Fernandez-Duque and Wifall (2007) reached the opposite conclusion for a task in which subjects chose from a set of ten cards (nine good cards and one bad card).

According to our findings, the prediction discrepancy under risk depends on the gain/loss domain and small/large probability: predictors are more risk-seeking than actors in large-probability gains and in small-probability losses, whereas predictors are less risk-seeking than actors in small-probability gains and in large-probability losses. Our results reconcile the conflicting findings in prior studies and suggest a significant role of contextual factors in the prediction discrepancy. Future research might focus on other contextual factors that are important in predicting others' decisions.

In our study, we used choice tasks to elicit risk preferences, in contrast to Faro and Rottenstreich (2006), who used pricing tasks in which participants were asked to indicate the certainty equivalent of a lottery. Although both pricing tasks and choice tasks have often been used to study risk preferences (Rottenstreich and Hsee, 2001; Gneezy et al., 2006; Sun et al., 2017a, 2018), studies have suggested differences between these two methodologies (Harbaugh et al., 2010). In addition, while we systematically considered different levels of probabilities in both the gain and loss domains, Faro and Rottenstreich (2006) used only lotteries with extreme probabilities (0.001 and 0.99) and investigated the effect of empathy gaps only in the gain domain. Therefore, our study greatly complements, and extends this study by Faro and Rottenstreich (2006).

One main contribution of our study is to show, using direct measures of emotional states, how the empathy gap is correlated with prediction discrepancy. While an empathy gap can influence a prediction by failing to fully appreciate the *intensity* or the *impact* of others' emotional states, our analysis implies that the empathy gap gives rise to prediction discrepancy mainly due to the underestimate of emotional intensity (not emotional impact). Additionally, we identified the types of emotions that influence prediction discrepancies under different conditions: with small- probability gains and large-probability losses, it is primarily the underestimated intensity of the anticipated elation that results in the prediction discrepancy; with large-probability gains and small-probability losses, it is mainly the underestimated intensity of the anticipated disappointment that plays the important role.

Before concluding, we want to point out some limitations of the current study. To explore the role of anticipated emotions, we asked participants to state their anticipated emotions prior to making their choices or predictions. This may suggest to participants that their emotions are important or should impact their selections. Because of the experimenter demand effect (Zizzo, 2010), it is possible that individuals use their stated emotions to assist their decision-making. Previous studies have shown that asking the participants to rate their emotional intensity does not significantly influence risky decision-making behavior (Sun, 2017), and the decisions and predictions in our study were consistent with previous studies where emotions were not elicited (Tversky and Kahneman, 1992; Faro and Rottenstreich, 2006). We therefore have some evidence that emotion elicitation does not significantly influence behaviors. However, we call for future studies without the potential influence of the experimenter demand effect to confirm the role of anticipated emotions in prediction discrepancy. Moreover, our paper only includes a single study with a moderate sample size, and related future studies are called for to confirm the conclusions in this paper.

Studies in neuroscience have demonstrated the neural mechanisms that cause the difference in decisions for oneself and decisions for others in a risky environment (Nicolle et al., 2012; Garvert et al., 2015; Suzuki et al., 2016). The exact mechanism behind prediction discrepancy still needs to be investigated. Although our study provides some indirect evidence on the importance of empathy gap in explaining the prediction discrepancy, and studies using neural techniques are necessary to discover the underlying mechanism and confirm the causal relationship.

To summarize, our study demonstrates the patterns of the prediction discrepancy in both gain and loss domains when different magnitudes of probabilities are present. We provide some evidence that such prediction discrepancy is highly correlated with the predictor's underestimate of the intensity (not the impact) of the actor's emotional state. From a practical perspective, understanding, and appreciating this prediction discrepancy can help individuals and firms to make better decisions in social and economic interactions; it can also help government and law enforcement to design and implement policies for the betterment of society.

## Author contributions

QS and HZ generating the ideas and design, writing and revising the article, and analysis of data. JZ design experimental materials, design computer programming, and collecting data. XZ meditation analysis of data, and collecting data.

### Conflict of interest statement

The authors declare that the research was conducted in the absence of any commercial or financial relationships that could be construed as a potential conflict of interest.

## Footnotes

^1^Power analysis based on the data from the first experimental session with 16 participants (8 actors and 8 predictors) shows that in order to detect a treatment effect in the RP index with the power of 0.8 and a significant level of 0.05 for all the four conditions, we need to 62 participants (31 for each role); in order to detect the difference in emotional intensity across all four conditions, at least 94 participants (48 for each role) are needed. Following previous studies on self-other differences in risky decision-making (Faro and Rottenstreich, 2006; Sun et al., 2017a), we ensured at least 60 participants in each condition in the current study.

^2^Previous studies have shown that people's behaviors can be altered by the act of observing or predicting others' risky decisions, and making decisions first can also influence predictions afterwards (Nicolle et al., 2012; Garvert et al., 2015; Suzuki et al., 2016). We choose a between-subject design to avoid this “order effect”.

^3^RMB is the currency used in China with the exchange rate of 1RMB = US$0.15 at the time of the experiment. Following most previous studies on this topic, we did not pay subjects on the basis of their choices. Studies have demonstrated that, although reducing the variance of results, incentives do not change the average behavior (Camerer, 1989; Tversky and Kahneman, 1992; Camerer and Hogarth, 1999).

^4^The RP index is essentially the same as the risk preference measure used by Suzuki et al. (2016)—they defined the risk preference as the proportion of gambles accepted relative to the proportion accepted by the risk-neutral agent, which can be obtained by subtract 0.5 (the proportion accepted by the risk-neutral agent) from RP index. Suzuki et al. (2016) show that this simple model-free measurement is consistent with other prevalent measurements based on utility functions.

^5^Tversky and Kahneman (1992) named *p* ≥ 0.5 as moderate and large probability. In this paper, we call it large probability for simplicity. Excluding *p* = 0.5 from the analysis gives almost identical results. Indeed, the Spearman correlation coefficients between the RP index of *p* = 0.5 and that of *p* > 0.5 is greater than 0.85 for both roles in both loss and gain domains.

^6^For the main effects, we also conducted non-parametric tests that are robust to the distributional skewness of the data and obtained identical results.

^7^The insignificant interaction terms show that predictors do not consistently underestimate the impact of the actor's emotional state. However, the significant coefficients of the variable “predictor” in Models (2) and (5) show that mis-estimation (not necessarily under-estimation) of emotional impact can explain some of the prediction discrepancy in the small probability conditions. Further studies are needed to investigate this.
